# Video consent significantly improves patient knowledge of general surgery procedures

**DOI:** 10.1007/s00464-024-10975-9

**Published:** 2024-06-26

**Authors:** Kristin Bremer, Emily Brown, Rachel Schenkel, Ryan W. Walters, Kalyana C. Nandipati

**Affiliations:** 1 Department of Surgery, Creighton University School of Medicine, Creighton University, 7710 Mercy Road, Education Building, Suite 501, Omaha, NE 68124 USA; 2 Department of Clinical Research and Public Health, Creighton University, Omaha, NE USA

**Keywords:** Consent, Video, Satisfaction, Appendectomy, Cholecystectomy, Fundoplication, Inguinal hernia

## Abstract

**Introduction:**

Informed consent is essential in ensuring patients’ understanding of their medical condition, treatment, and potential risks. The objective of this study was to investigate the impact of utilizing a video consent compared to standard consent for patient knowledge and satisfaction in selected general surgical procedures.

**Methods and procedures:**

We included 118 patients undergoing appendectomy, cholecystectomy, inguinal hernia repair, and fundoplication at two hospitals in Omaha, NE. Patients were randomized to either a standard consent or a video consent. Outcomes included a pretest and posttest objective knowledge assessment of their procedure, as well as a satisfaction survey which was completed immediately after consent and following discharge. Given the pre-post design, a linear mixed-effect model was estimated for both outcomes. A two-way interaction effect was of primary interest to assess whether pre-to-post change in the outcome differed between patients randomized to standard or video consent.

**Results:**

Baseline characteristics were mostly similar between groups except for patient sex, *p* = 0.041. Both groups showed a statistically significant increase in knowledge from pretest to posttest (standard group: 0.25, 95% CI 0.01 to 0.51, *p* = 0.048; video group: 0.68, 95% CI 0.36 to 1.00, *p* < 0.001), with the video group showing significantly greater change (interaction *p* = 0.043) indicating that incorporating a video into the consent process resulted in a better improvement in patient’s knowledge of the proposed procedure. Further, both groups showed a decrease in satisfaction post-discharge, but no statistically significant difference in the magnitude of decrease between the groups (interaction *p* = 0.309).

**Conclusion:**

Video consent lead to a significant improvement in a patient’s knowledge of the proposed treatment. Although the patient satisfaction survey didn’t show a significant difference, it did show a trend. We propose incorporating videos into the consent process for routine general surgical procedures.

**Supplementary Information:**

The online version contains supplementary material available at 10.1007/s00464-024-10975-9.

Informed consent is a core component of ethical care and requires ensuring that a patient understands their medical condition, the natural course of the disease, a discussion of the proposed treatment, risks and common complications, the expected benefit of treatment, alternative treatment strategies, and the medical staff who will be involved in their care [1]. A truly informed consent requires not just a discussion, but an understanding of the discussion on the part of the patient or their representative [2]. This is more difficult to ensure, as many patients cannot fully understand all the risks and alternatives to a medical procedure because they lack sufficient medical knowledge and experience. Studies have demonstrated concerning trends in patients’ understanding of the procedure they have consented to undergo [3]. In some studies, patients averaged a 50% correct response rate on knowledge questionnaires [4]. Other studies reported that less than half of patients could name their surgeon, list a single complication of their operation, or felt that they adequately understood their proposed treatment [3]. Inadequate knowledge of the procedure can potentially lead to mistrust between the patient and physician, which may also impact patient satisfaction and medical litigation. It is imperative for physicians, and the health system, to ensure patients' knowledge and a truly informed consent, which may also lead to improved patient satisfaction and overall outcomes.

Several ideas have been used in the past to improve a patient’s knowledge including handouts, slides, and other multimedia presentations with mixed results [3–5]. Many multimedia projects used video to convey information across various specialties [5–14]. Most studies suggest an improvement in understanding of the proposed procedure as measured by questionnaire responses [6, 12, 13]. Various factors have been reported to influence scores on these questionnaires, including prior healthcare experience, age, and educational attainment [13]. Zhang et. al. found that the video presentation seemed to be more impactful for those patients with lower educational attainment [12], while Saglam found that the video presentation led to an equalization of questionnaire scores between those without a university degree to those with a university degree [13]. These video presentations can be a significant way for the medical team to improve a patient, as well as family member’s, understanding of a proposed procedure, while at the same time requiring less of the surgeon’s time to impart this information [6, 8]. For patients with electively scheduled procedures, there also exists an opportunity to review videos outside of the clinical environment so that the discussion with the surgeon can focus more on their specific questions or concerns [5].

The impact of video-based education on general surgical procedures, such as appendectomies, cholecystectomies, and hernia repairs, across clinic and hospital-based settings has not been well studied. This presents an intriguing opportunity to investigate the impact of integrating video into the consent procedure for these surgeries. The aim of the current study was to investigate whether the addition of a fully illustrated video to the surgical consent process led to improved patient knowledge, measured by a procedure specific questionnaire, and its impact on overall patient satisfaction with a validated satisfaction questionnaire.

## Materials and methods

**Fig. 1:**
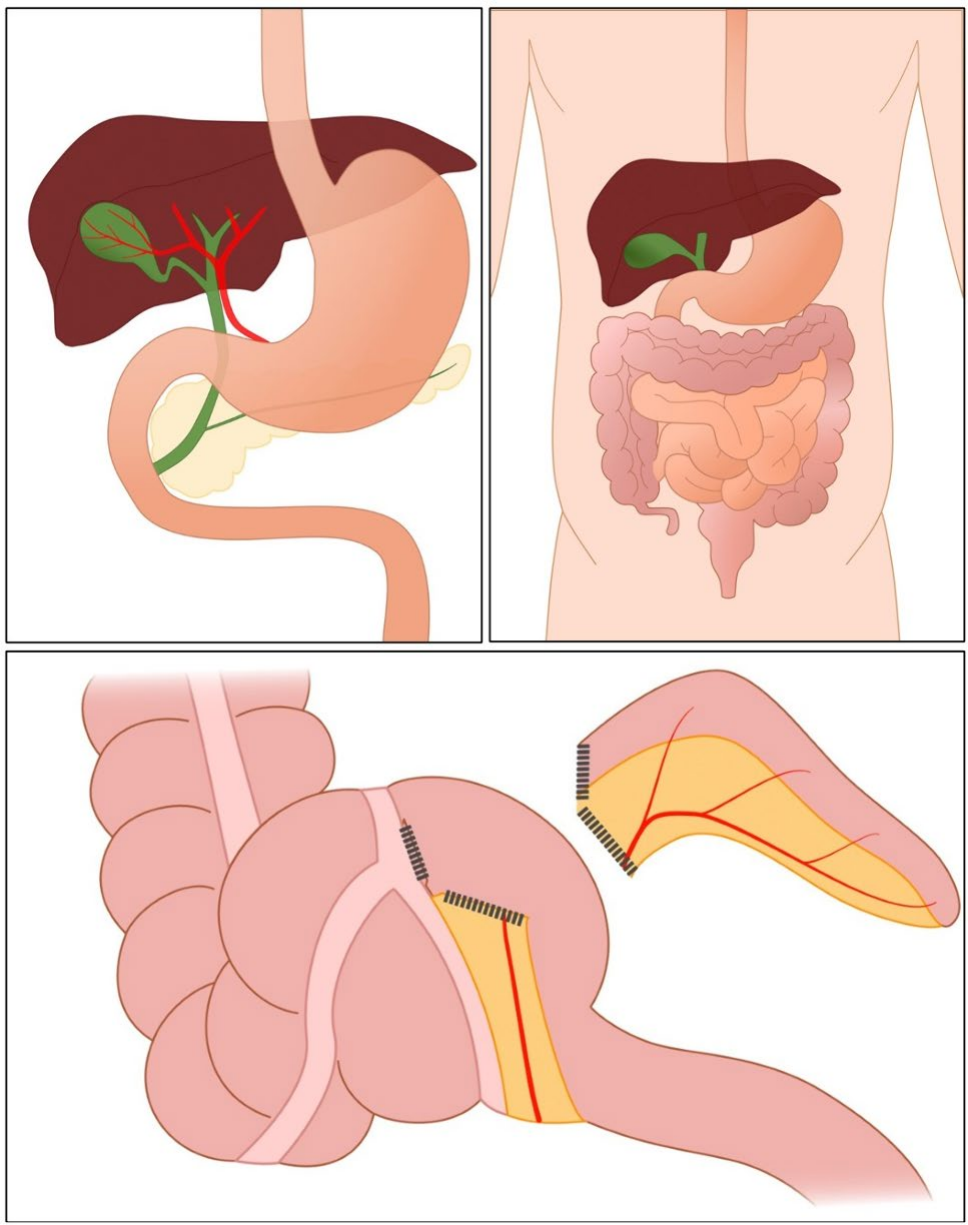
Examples of illustrations used in consent videos Credit: Kristin Bremer, MD

This was a randomized controlled trial that included patient encounters in both the ambulatory and inpatient settings at two hospitals, with one 396-bed university-based hospital (Creighton University Medical Center at Bergan Mercy) and another 352-bed university affiliated community medical center (Immanuel Medical Center) in Omaha, Nebraska. We created comprehensive video surgical consents for each of the following operations: laparoscopic appendectomy, minimally invasive fundoplication (laparoscopic and robotic), laparoscopic cholecystectomy, and inguinal hernia repair by any approach. These consent videos were designed to educate patients on the basics of how these operations are performed and the risks, benefits, and alternatives for each surgery. These were fully illustrated videos, with illustrations done by one of the study authors. The videos averaged 7 min and 4 s. Affinity Designer was used on an iPad to create the illustrations. Lightworks was used on a Windows laptop to create each video. Example illustrations are provided in Fig. 1.

The process for both groups began with informed consent for participation in the study and a release of information consent. Randomization of patients to the video consent versus standard surgical consent groups was accomplished by flipping a coin. Both groups then completed a pre-test knowledge survey for their respective procedure. The patients in the standard consent group consented with an in-person verbal discussion with a surgical resident and/or attending, while the patients in the video group watched the video specific to their procedure followed by a similar in-person verbal discussion. As part of the standard consent, individual surgeons or residents could elect to draw anatomic pictures if this was part of their typical consent discussion. After this, both groups had an opportunity to have any questions answered.

**Fig. 2:**
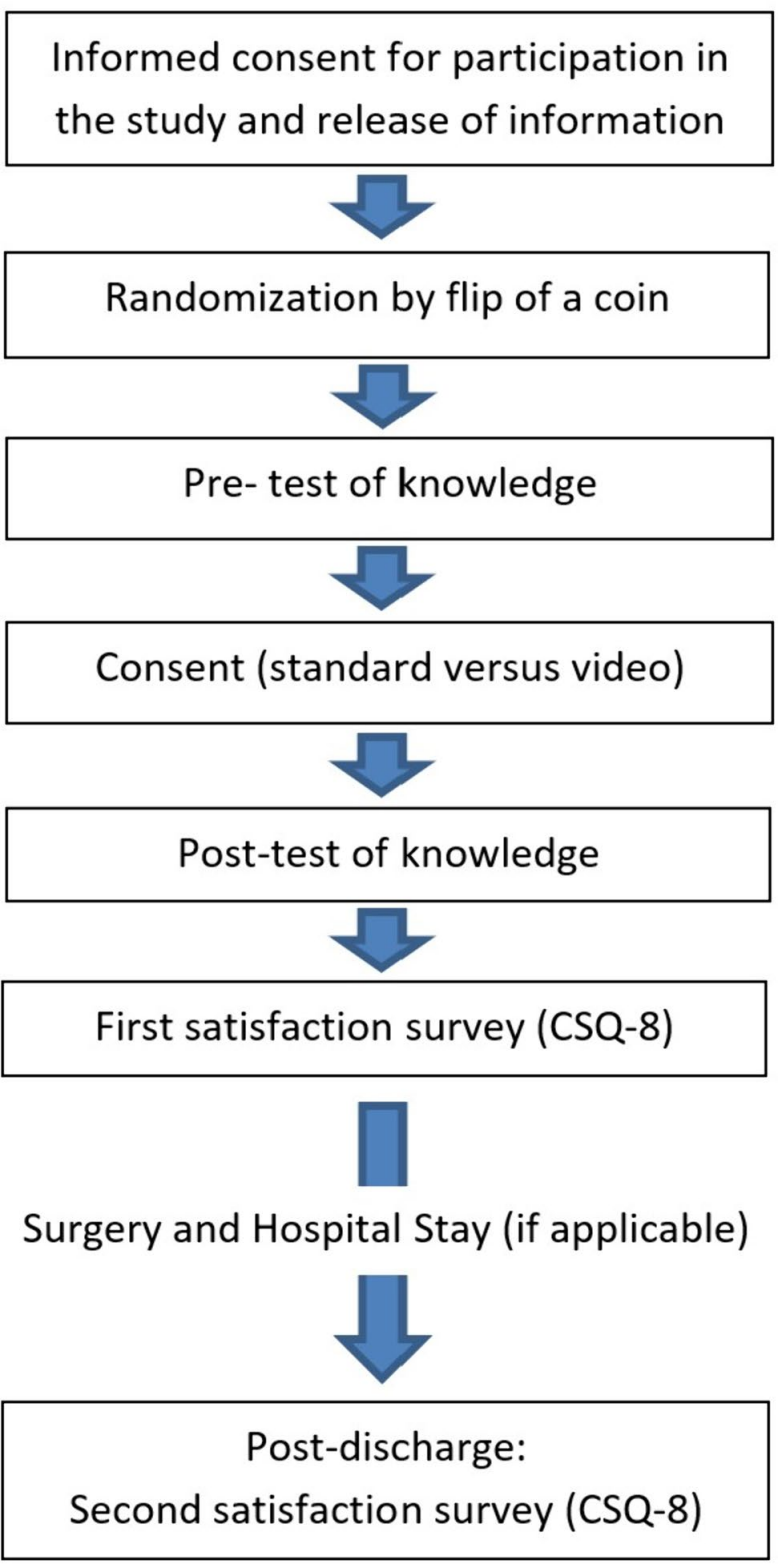
Workflow illustration

Patients then completed a post-test survey of knowledge for their respective procedure as well as a CSQ-8 patient satisfaction survey. Following this, patients proceeded with their planned surgery and hospital stay, if applicable. Patients completed an additional CSQ-8 patient satisfaction survey following discharge. These were either collected when the patient returned to the clinic for post-operative follow-up or were administered by telephone by research assistants. Very few patients mailed their response. The surgical consent workflow as described above is illustrated in Fig. 2. This workflow was typically done in the clinic setting at the patients’ preoperative visit for outpatient procedures or in the emergency room, inpatient room, or preoperative suite for patients who presented acutely to the hospital.

The CSQ-8 satisfaction survey is a standard patient satisfaction survey that is not specific to the consent process, but rather the overall patient experience. We were unable to find a validated survey for the patient tests of knowledge, so one was created for each procedure with six questions each. An effort was made to avoid directly mirroring what was covered in the videos, and to include only things that would be expected to be covered during a thorough consent conversation, such as basic anatomy, surgical indications, risks, benefits, alternatives, and postoperative expectations. Questionnaires were provided in appendix.

The change in knowledge survey score after consent was calculated for each patient and utilized to compare the two study groups. Satisfaction scores were also calculated both at the immediate post-consent time as well as post-discharge. The surgeons and research personnel were not blinded to the contents of the video, the tests of knowledge, or the patient satisfaction surveys.

Patient Demographics that were collected included the patient’s age, sex, race/ethnicity, and history of prior abdominal surgery. Only those individuals over the age of 19 were included, as this is the age of consent in the State of Nebraska. Furthermore, patients had to undergo one of the following procedures during the study period: Laparoscopic Appendectomy, Laparoscopic Cholecystectomy, Inguinal Hernia (laparoscopic/robotic/open), and Fundoplication (laparoscopic/robotic). Patients who were unable to provide consent for themselves or who required the use of a translator for a primary language other than English were excluded.

All descriptive statistics are stratified by consent group (standard vs. video). Continuous variables are presented as mean and standard deviation, compared using independent-samples t-test. Categorical variables are presented as count and percent, compared using the chi-square test or Fisher's exact test. Changes in knowledge and satisfaction were compared using linear mixed effects models with a random intercept to account for correlation inherent to observations from the same patient. Heterogenous variances were allowed for each consent group and measurement point. The fixed effects of interest were the time-by-group interaction to determine whether change in each outcome differed by group. We also evaluated a three-way interaction effect between time, group, and consent location (clinic vs. ED). All analyses were conducted using SAS v. 9.4 with two-tailed *p* < 0.05 indicating statistical significance.

## Results

**Fig. 3:**
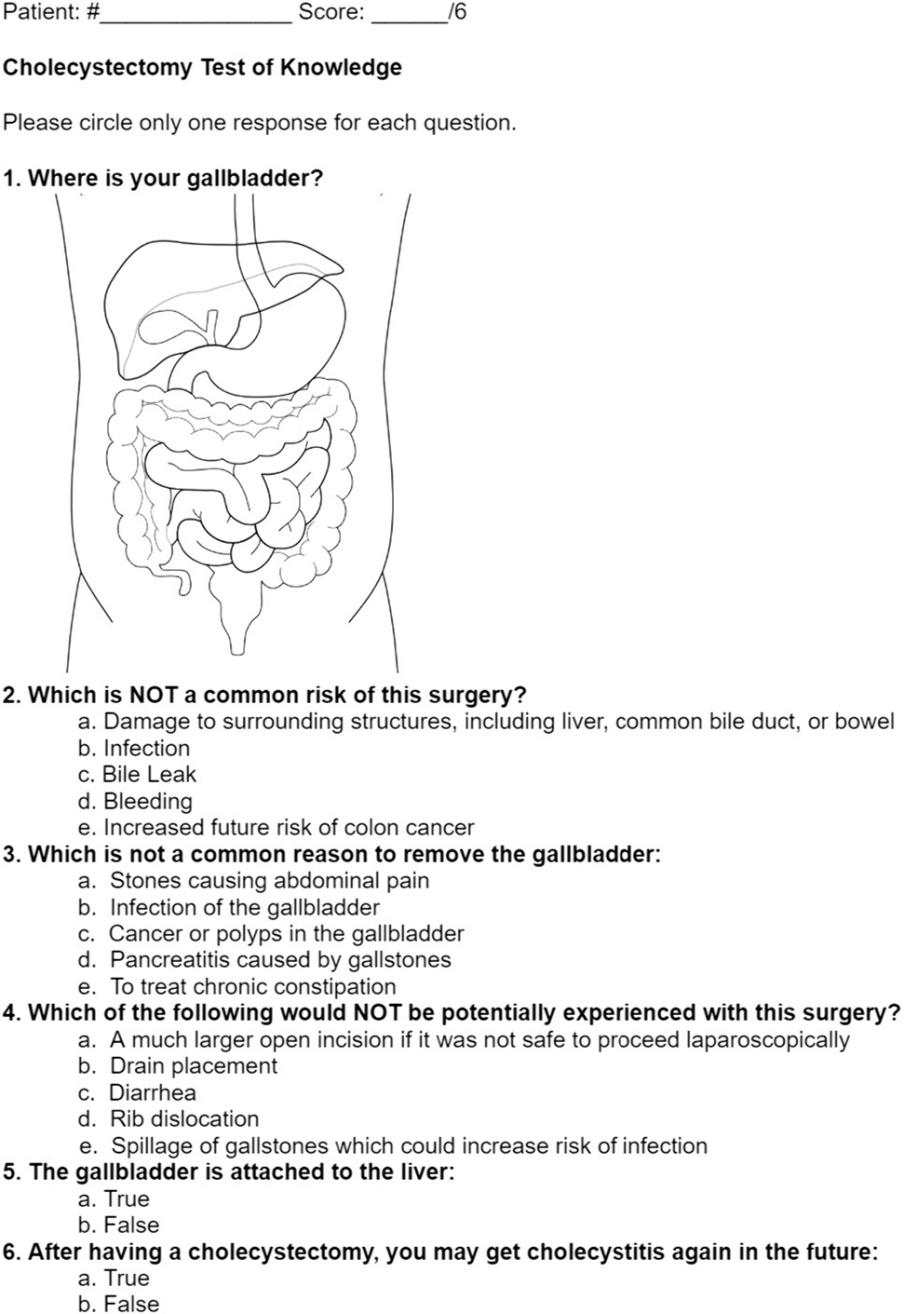
Example test of knowledge

In total 118 patients were included in the study (timeline), of whom 17 (14.4%) underwent appendectomy, 34 (28.8%) underwent cholecystectomy, 53 (44.9%) underwent fundoplication, and 14 (11.9%) underwent inguinal hernia repair. Consent was obtained in the clinic for 79 patients (66.9%), in the emergency department for 36 patients (30.5%), and in an inpatient room for 3 patients (2.5%). A total of 59 patients (50%) were randomized to the standard group and the rest were randomized to the video consent group. The demographic and clinical characteristics stratified by consent method are shown in Table 1. Despite randomization, there was a statistically significant difference in sex between the groups, with more women in the standard consent group and more men in the video consent group (*p* = 0.041). There were no other statistically significant differences in demographic or clinical characteristics between the groups (Fig. 3).

**Table 1:** Demographic and clinical characteristics

	Standard	Video	*p*
Age	52.1 ± 12.4	52.4 ± 17.8	0.928
Biological sex			
Female	39 (66.1)	28 (47.5)	0.041
Male	20 (33.9)	31 (52.5)	
Race			
White	49 (83.1)	44 (74.6)	0.461
Black	5 (8.5)	7 (11.9)	
Hispanic/Latino	1 (1.7)	4 (6.8)	
Other	1 (1.7)	2 (3.4)	
Missing	3 (5.1)	2 (3.4)	–
Prior abdominal surgery	16 (27.1)	11 (18.6)	0.273
Procedure			
Appendectomy	11 (18.6)	6 (10.2)	0.268
Cholecystectomy	14 (23.7)	20 (33.9)	
Fundoplication	29 (49.2)	24 (40.7)	
Inguinal hernia repair	5 (8.5)	9 (15.3)	
Consent obtained			
Clinic	38 (64.4)	41 (69.5)	0.658
Emergency department	20 (33.9)	16 (27.1)	
Patient room	1 (1.7)	2 (3.4)	

Data presented as mean ± SD or count (%)

**Fig. 4:**
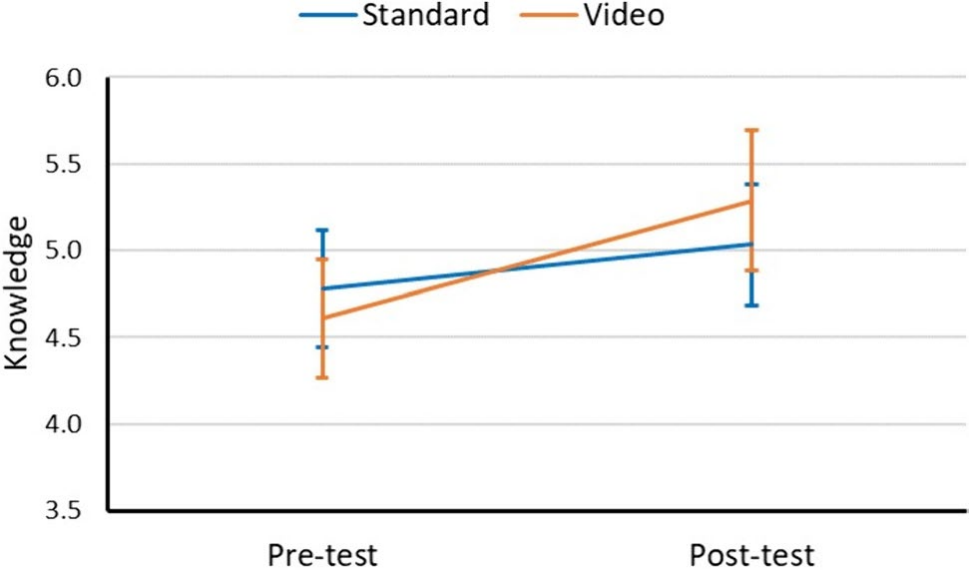
Change in knowledge scores stratified by consent group. Error bars represent 95% confidence intervals

There was no difference in knowledge scores pre-consent (standard: 4.78 vs. video: 4.61; *p* = 0.485; Fig. 4). Although knowledge of both groups increased pre-consent to post-consent (standard: 0.25, 95% CI 0.01 to 0.51, *p* = 0.048 vs. video: 0.68, 95% CI 0.36 to 1.00, *p* < 0.001), knowledge scores improved to a greater extent in the video consent group compared to the standard consent group (time-by-group interaction *p* = 0.043; change difference: 0.42, 95% CI 0.01 to 0.83). There was not a statistically significant between-group difference post-consent (standard: 5.03 vs. video: 5.29, *p* = 0.347). When the groups were further stratified by location of consent (i.e., clinic vs. emergency department), both consent groups showed statistically similar change (time-by-group-by-location interaction *p* = 0.681; Table 2). The vast majority of encounters were in the clinic or emergency department, with only a few occurring in inpatient rooms or the preoperative suite. Knowledge scores showed a statistically significant improvement for patients consented in the clinic, but not for patients consented in the ED; this was likely a function of the different sample sizes by location of consent.

**Table 2:** Pre-to-post consent change in knowledge scores

Consent location	Pre-consent			Post-consent			Change from pre-consent to post-consent				Interaction *p*
	Standard	Video	*p*	Standard	Video	*p*	Standard	Video	Difference (95% CI)	*p*	
Clinic	4.76	4.80	0.888	4.92	5.46	0.099	0.16	0.66	0.50 (0.01 to 1.00)	0.049	0.681
ED	4.75	4.19	0.205	5.25	5.00	0.612	0.50	0.81	0.31 (– 0.44 to 1.07)	0.414	

Interaction *p* indicates whether the pre-to-post change in knowledge between standard and video differed by whether consent was obtained in the clinic or emergency department

**Fig. 5:**
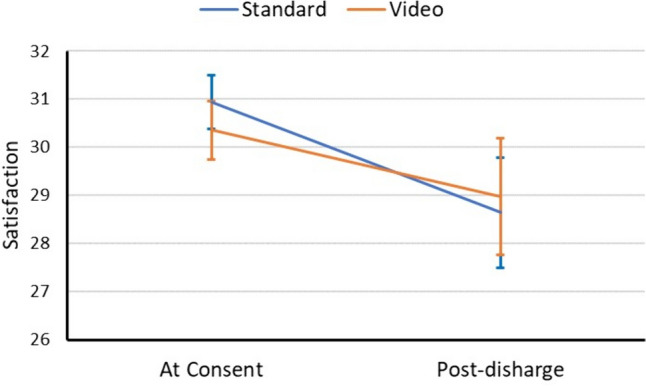
Change in satisfaction scores stratified by consent group. Error bars represent 95% confidence intervals

There was no difference in satisfaction scores immediately after consent (standard: 30.93 vs. video: 30.36; *p* = 0.170; Fig. 5). Both groups showed decreased satisfaction at consent to post-discharge follow-up (standard: – 2.29, 95% CI – 3.48 to – 1.09, *p* < 0.001 vs. video: – 1.38, 95% CI –2.67 to – 0.10, *p* = 0.036). Although the video consent group showed less of a decrease in satisfaction, there was no difference in change between the two consent groups (time-by-group interaction *p* = 0.309); there was not a statistically significant between-group difference post-discharge (standard: 28.64 vs. video: 28.97, *p* = 0.697). When the groups were stratified by location of consent, both consent groups showed statistically similar change (time-by-group-by-location interaction *p* = 0.510; Table 3); change for both groups was not statistically significant.

**Table 3:** Satisfaction scores immediately after consent and post-discharge

Consent location	Immediately after consent			Post-discharge			Change from at consent to post-discharge				Interaction *p*
	Standard	Video	*p*	Standard	Video	*p*	Standard	Video	Difference (95% CI)	*p*	
Clinic	31.47	30.44	0.035	29.18	29.50	0.776	– 2.29	– 0.94	– 1.34 (– 3.63 to 0.93)	0.244	0.510
ED	29.85	30.44	0.421	27.70	28.38	0.636	– 2.15	– 2.06	– 0.09 (– 3.10 to 2.93)	0.954	

Interaction *p* indicates whether the after consent to post-discharge change in satisfaction between standard and video differed by whether consent was obtained in the clinic or emergency department

## Discussion

The current study’s results showed a statistically significant increase in knowledge scores for the video consent group compared to the standard consent group. Similarly, literature published across other disciplines also showed improved understanding of the proposed procedure as measured by questionnaire responses [1, 10, 11]. This suggests that patients in the video consent group were better informed about the surgical procedure they were undergoing, including procedural anatomy, steps, risks, benefits, and alternatives. Video-based consent process has several inherent advantages. These video presentations not only provide a better way for the medical team to improve a patient’s understanding of a proposed procedure, but also requires less of the surgeon’s time to impart this education [6, 8]. It also provides opportunity for patient to share information with their support system who may or may not physically present for the discussion with the surgeon [9]. For patients with electively scheduled procedures, they may also review videos outside of the clinical environment so that the discussion with the surgeon can focus more on their specific questions or concerns [5]. Although these consequences of video consent were not specifically studied, many participants were observed to ask more detailed and in-depth questions when the surgeon was present after watching the video.

Our results only showed knowledge improvement in overall study population but failed to show any significant difference in the subgroup analysis involving patients consented in the emergency department. The most important reason behind lack of significant difference was due to insufficient power in each group, especially from the emergency department. It is possible that the setting may have impacted the result, however, an interaction p-value was calculated to investigate this question and it was not statistically significant. Other factors that may potentially influence this finding include the amount of time spent on the consent process, though this was not investigated in this study. Patients consenting in the emergency department may also be under increased stress as they are likely consenting to a more urgent surgical procedure and experiencing acute pain at the time of consent. As suggested by McKeague and colleagues, patients may also feel more social pressure to complete the consent process in a timely matter and may agree to proceed before feeling they have a sufficient understanding [3]. This may be mitigated in the clinic if they feel less rushed going through the consent process.

Some prior studies have raised concerns about how detailed presentations and videos may affect a patient’s anxiety about an upcoming procedure. While a patient’s anxiety is not a valid reason to neglect an appropriate informed consent process, it is logical that not every patient wants the same level of detail or to see images or videos of the procedure itself. Most studies however [5, 10, 12]. have not demonstrated any significant increase in a patient’s anxiety after viewing a video as part of their consent process. Similarly, several studies have attempted to measure patient satisfaction with multimedia or video consent. One study found no significant difference in patient satisfaction, which they interpreted as the videos did not decrease patient satisfaction [6]. However, Zhang et al. reported higher patient satisfaction [8] but noted that their patient population was allowed to watch the videos as many times as they desired while Mawhinney and colleagues also found improved satisfaction and noted that their patients often watched the videos with family or friends [9].

Many multimedia projects have focused on video to convey information [4–7, 9–13] across various specialties. The ways in which these videos are distributed vary widely: some are viewed by patients on their own time, some pre-operatively in the clinic, and some in the pre-operative holding area. Overall, most studies seem to suggest that patients viewing a video as part of the consent process have an improved understanding about the procedure, similar to our study. Several factors have been noted to influence scores on these questionnaires, including prior healthcare experience, age, and educational attainment [13]. Patient demographics had limited impact in our study, but prior health care experience may have played a significant role in knowledge improvement and overall satisfaction. Zhang et al. found that the video presentation seemed to be more impactful for those patients with lower educational attainment [12], while Saglam found that the video presentation led to an equalization of questionnaire scores between those without a university degree to those with a university degree [13]. Our study is a randomized controlled trial which may have limited the impact of patient demographics and educational background. One inherent limitation of the study was not evaluating the patient educational attainment in the analysis.

There were no statistically significant differences in overall patient satisfaction between the groups. Though we had hypothesized that the video group would be more satisfied with the consent process, our satisfaction survey was not specific to the consent process but rather to their entire clinical encounter. This introduced an immense amount of confounding factors, including their interactions with staff in the clinic, emergency department, operating room, recovery room, and (if applicable) on the inpatient ward. This is further suggested by the decline in patient satisfaction for both groups on the post-discharge survey. Though there was no comment section on the satisfaction survey, a few patients who had given particularly poor scores wrote in the margins about various negative experiences they had experienced during their hospitalization. Overall, both groups demonstrated a decline in patient satisfaction after hospitalization, the trend in the standard consent group was toward a larger decrease in satisfaction. There are many challenges, particularly in the post-COVID environment, that may have influenced this finding, particularly more limited staffing. Despite limited hospital resources, physician reimbursement is still somewhat tied to overall patient satisfaction. The video consent process may help physicians to prevent larger drop in patient satisfaction, though this study did not have sufficient power to confirm this.

This randomized control trial was limited in many ways by small sample sizes, a lack of blinding, and being conducted only at two institutions. It is possible that with a higher-powered study, statistically significant changes in patient satisfaction and/or changes in knowledge for patients consented in different clinical environments may have become more apparent. Other limitations included a lack of blinding, exclusion of non-English speaking patients, and limits to generalizability given that it only involved two institutions within a single city with a predominantly white population. Like other studies, our tests of knowledge had not been previously validated [5]. This raises questions of whether the questions were suitable for the average patient. We did not control for the patient’s prior educational attainment or exposure to the procedure, which may also influence their ability to learn and understand the medical terminology presented during the consent process. Similarly, we did not control for income.

We believe adding video to the consent process adds value in helping improve patient’s understanding of their proposed treatment. We also believe additional study focused on the impact of adding video to the time required for the consent process, and if this may impact the efficiency of a patient’s care, would be valuable. If video consent requires significantly less time, while simultaneously improving patient’s understanding, this may also improve physician burnout by providing opportunities for physicians to have more meaningful and patient-centric discussions.

## Conclusion

Incorporating a video into the consent process can lead to a significant improvement in a patient’s knowledge of the proposed treatment. Although the patient satisfaction survey did not show a significant difference, it did show a trend toward a smaller reduction in satisfaction for the video group. Video consent process could serve as a valuable supplement to the consent process for several general surgical procedures.

### Supplementary Information

Below is the link to the electronic supplementary material.

Supplementary file1 (JPG 208 kb)

Supplementary file2 (MP4 102969 kb)

Supplementary file3 (MP4 139927 kb)

Supplementary file4 (JPG 210 kb)

Supplementary file5 (MP4 147187 kb)

Supplementary file6 (JPG 234 kb)

Supplementary file7 (MP4 140715 kb)

## References

[1] American College of Surgeons (2016) Statements on principles. https://www.facs.org/about-acs/statements/statements-on-principles/#anchor171960

[2] Shah P, Thornton I, Turrin D et al (2023) Informed consent. In: StatPearls [Internet]. Treasure Island (FL): StatPearls Publishing. Available from: https://www.ncbi.nlm.nih.gov/books/NBK430827/# 28613577

[3] McKeague M, Windsor J (2003) Patients’ perception of the adequacy of informed consent: a pilot study of elective general surgical patients in Auckland. N Z Med J 116(1170):U355 12658314

[4] Mulsow JJ, Feeley TM, Tierney S (2012) Beyond consent–improving understanding in surgical patients. Am J Surg 203(1):112–120. 10.1016/j.amjsurg.2010.12.010 21641573 10.1016/j.amjsurg.2010.12.010

[5] Nehme J, El-Khani U, Chow A, Hakky S, Ahmed AR, Purkayastha S (2013) The use of multimedia consent programs for surgical procedures: a systematic review. Surg Innov 20(1):13–23. 10.1177/1553350612446352 22589017 10.1177/1553350612446352

[6] Pallett AC, Nguyen BT, Klein NM, Phippen N, Miller CR, Barnett JC (2018) A randomized controlled trial to determine whether a video presentation improves informed consent for hysterectomy. Am J Obstet Gynecol 219(3):277.e1-277.e7. 10.1016/j.ajog.2018.06.016 29959929 10.1016/j.ajog.2018.06.016

[7] Writing Group for the CODA Collaborative (2023) A video-based consent tool: development and effect of risk-benefit framing on intention to randomize. J Surg Res 283:357–367. 10.1016/j.jss.2022.10.089 36427446 10.1016/j.jss.2022.10.089

[8] Zhang Y, Ruan X, Tang H, Yang W, Xian Z, Lu M (2017) Video-assisted informed consent for cataract surgery: a randomized controlled trial. J Ophthalmol 2017:9593631. 10.1155/2017/9593631 28191349 10.1155/2017/9593631 PMC5278206

[9] Mawhinney G, Thakar C, Williamson V, Rothenfluh DA, Reynolds J (2019) Oxford video informed consent tool (OxVIC): a pilot study of informed video consent in spinal surgery and preoperative patient satisfaction. BMJ Open 9(7):e027712. 10.1136/bmjopen-2018-027712 31345967 10.1136/bmjopen-2018-027712 PMC6661683

[10] Sahai A, Kucheria R, Challacombe B, Dasgupta P (2006) Video consent: a pilot study of informed consent in laparoscopic urology and its impact on patient satisfaction. JSLS 10(1):21–25 16709351 PMC3015669

[11] Penn JP, Nallani R, Dimon EL, Daniels TC, Sykes KJ, Chiu AG, Villwock MR, Villwock JA (2021) Educational informed consent video equivalent to standard verbal consent for Rhinologic surgery: a randomized controlled trial. Am J Rhinol Allergy 35(6):739–745. 10.1177/1945892421992659 33530693 10.1177/1945892421992659 PMC9793427

[12] Zhang MH, Haq ZU, Braithwaite EM, Simon NC, Riaz KM (2019) A randomized, controlled trial of video supplementation on the cataract surgery informed consent process. Graefes Arch Clin Exp Ophthalmol 257(8):1719–1728. 10.1007/s00417-019-04372-5 31144057 10.1007/s00417-019-04372-5 PMC7846231

[13] Saglam K, Kayaalp C, Aktas A, Sumer F (2020) Educational video addition to the bariatric surgery informed consent process: a randomized controlled trial. Obes Surg 30(7):2693–2699. 10.1007/s11695-020-04552-x 32279184 10.1007/s11695-020-04552-x

[14] Lin YK, Chen CW, Lee WC, Cheng YC, Lin TY, Lin CJ, Shi L, Tien YC, Kuo LC (2018) Educational video-assisted versus conventional informed consent for trauma-related debridement surgery: a parallel group randomized controlled trial. BMC Med Ethics 19(1):23. 10.1186/s12910-018-0264-7 29523129 10.1186/s12910-018-0264-7 PMC5845218

