# Supplementary figures and images for: Video consent significantly improves patient knowledge of general surgery procedures
Source: Surg Endosc. 2024 Jun 26;38(8):4641–7. doi: 10.1007/s00464-024-10975-9 (PMC11289049; doi:10.1007/s00464-024-10975-9)

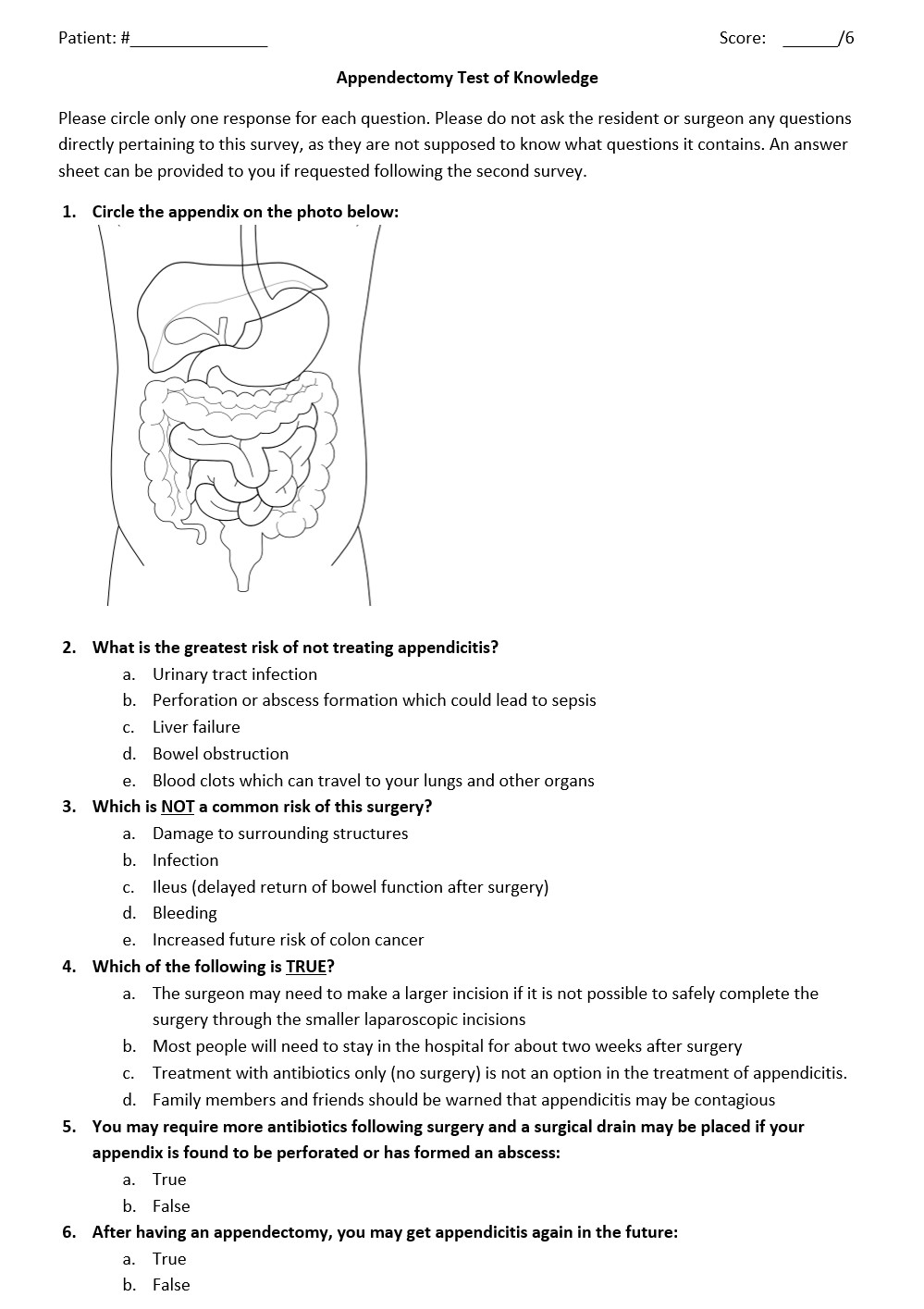

Supplement: Supplementary file 1 — Supplementary file1 (JPG 208 kb) [file 464_2024_10975_MOESM1_ESM.jpg]

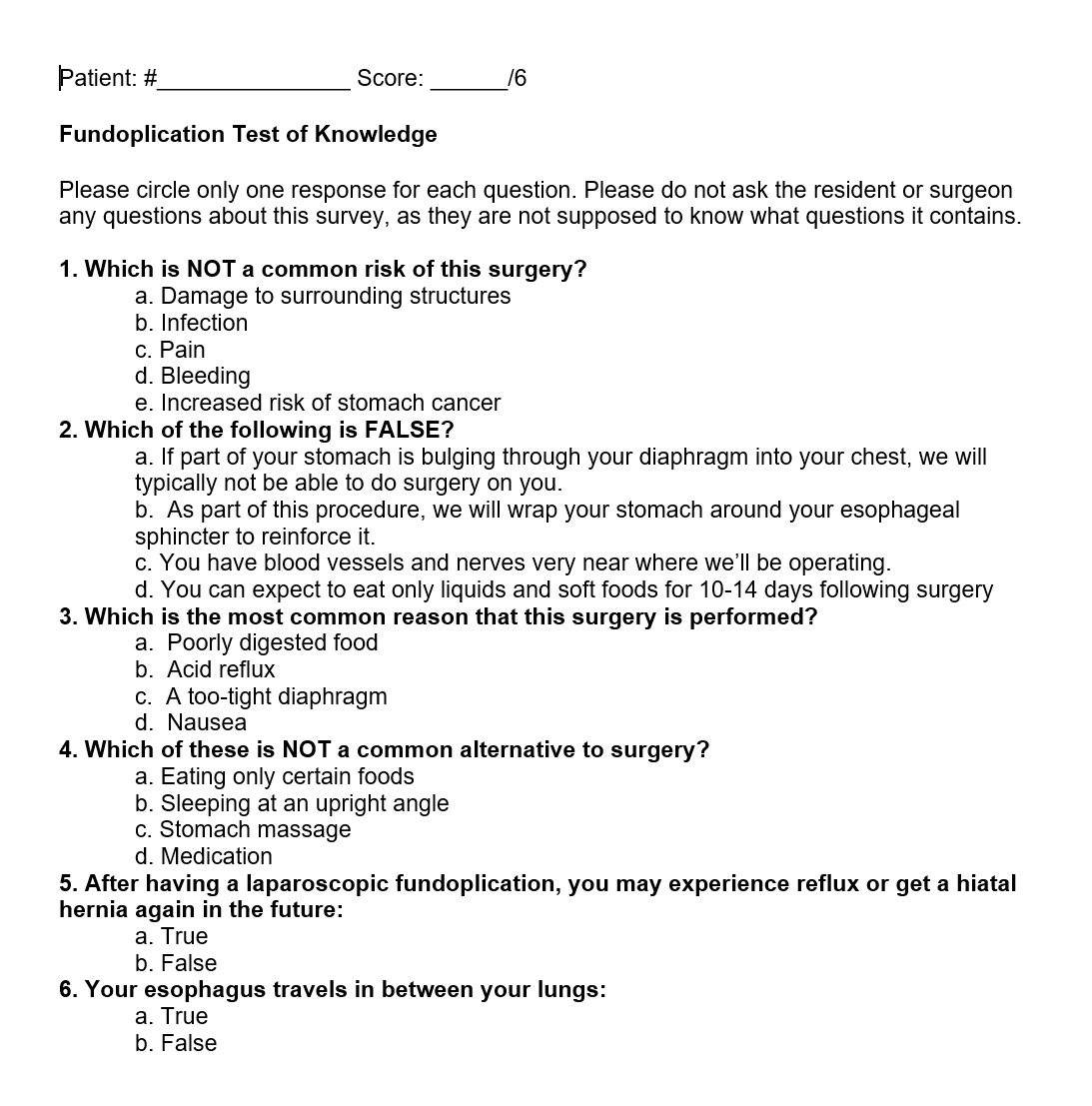

Supplement: Supplementary file 4 — Supplementary file4 (JPG 210 kb) [file 464_2024_10975_MOESM4_ESM.jpg]

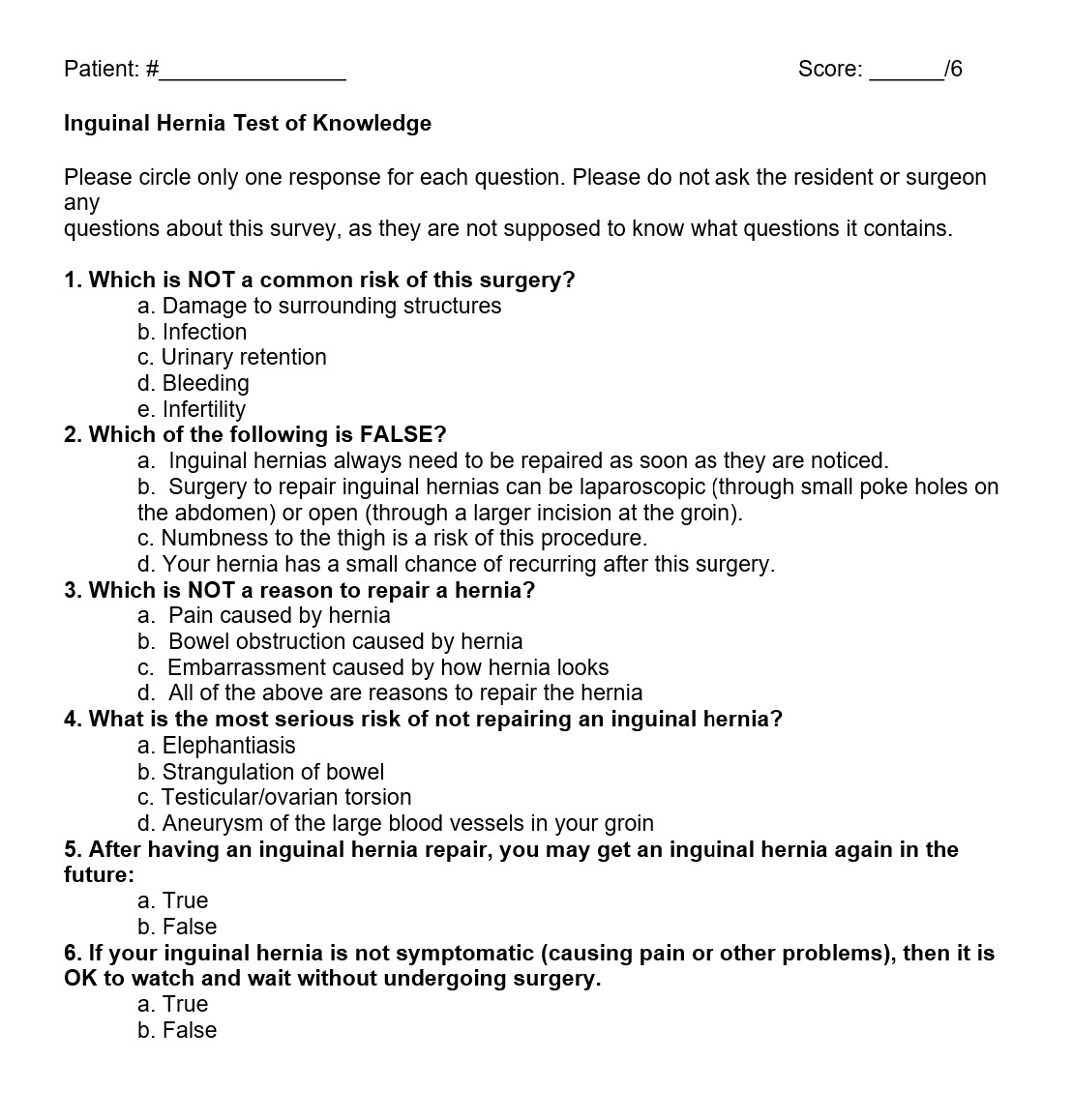

Supplement: Supplementary file 6 — Supplementary file6 (JPG 234 kb) [file 464_2024_10975_MOESM6_ESM.jpg]
